# Pseudomonads Rule Degradation of Polyaromatic Hydrocarbons in Aerated Sediment

**DOI:** 10.3389/fmicb.2015.01268

**Published:** 2015-11-19

**Authors:** Jiri Wald, Miluse Hroudova, Jan Jansa, Blanka Vrchotova, Tomas Macek, Ondrej Uhlik

**Affiliations:** ^1^Department of Biochemistry and Microbiology, Faculty of Food and Biochemical Technology, University of Chemistry and TechnologyPrague, Prague, Czech Republic; ^2^Department of Genomics and Bioinformatics, Institute of Molecular Genetics, Czech Academy of SciencesPrague, Czech Republic; ^3^Laboratory of Fungal Biology, Institute of Microbiology, Czech Academy of SciencesPrague, Czech Republic

**Keywords:** naphthalene, polyaromatic hydrocarbons, biodegradation, stable isotope probing, dioxygenase, *Pseudomonas veronii*, *Pseudomonas gessardii*, *Comamonas testosteroni*

## Abstract

Given that the degradation of aromatic pollutants in anaerobic environments such as sediment is generally very slow, aeration could be an efficient bioremediation option. Using stable isotope probing (SIP) coupled with pyrosequencing analysis of 16S rRNA genes, we identified naphthalene-utilizing populations in aerated polyaromatic hydrocarbon (PAH)-polluted sediment. The results showed that naphthalene was metabolized at both 10 and 20°C following oxygen delivery, with increased degradation at 20°C as compared to 10°C—a temperature more similar to that found *in situ*. Naphthalene-derived ^13^C was primarily assimilated by pseudomonads. Additionally, *Stenotrophomonas, Acidovorax, Comamonas*, and other minor taxa were determined to incorporate ^13^C throughout the measured time course. The majority of SIP-detected bacteria were also isolated in pure cultures, which facilitated more reliable identification of naphthalene-utilizing populations as well as proper differentiation between primary consumers and cross-feeders. The pseudomonads acquiring the majority of carbon were identified as *Pseudomonas veronii* and *Pseudomonas gessardii*. Stenotrophomonads and *Acidovorax defluvii*, however, were identified as cross-feeders unable to directly utilize naphthalene as a growth substrate. PAH degradation assays with the isolated bacteria revealed that all pseudomonads as well as *Comamonas testosteroni* degraded acenaphthene, fluorene, and phenanthrene in addition to naphthalene. Furthermore, *P. veronii* and *C. testosteroni* were capable of transforming anthracene, fluoranthene, and pyrene. Screening of isolates for naphthalene dioxygenase genes using a set of in-house designed primers for Gram-negative bacteria revealed the presence of such genes in pseudomonads and *C. testosteroni*. Overall, our results indicated an apparent dominance of pseudomonads in the sequestration of carbon from naphthalene and potential degradation of other PAHs upon aeration of the sediment at both 20 and 10°C.

## Introduction

Polyaromatic hydrocarbons (PAHs) are ubiquitous pollutants of either anthropogenic or natural origin (Shuttleworth and Cerniglia, 1995). PAHs of anthropogenic origin are typically derived from fossil fuel combustion, refining of crude oil, tar production, waste incineration, and food technologies, whereas naturally derived PAHs originate from forest fires, natural oil seeps, and some microbial processes. Several microorganisms, including bacteria, yeasts, and other fungi, have been implicated in the metabolism of PAHs (Cerniglia, 1992; Pothuluri and Cerniglia, 1994; Cerniglia and Sutherland, 2010). Low molecular weight PAHs with up to three rings, such as naphthalene, phenanthrene, and anthracene, can serve as carbon sources for many bacteria whose complete degradation pathways have been well-described (van der Meer et al., 1992). However, it is less common for microorganisms to degrade higher molecular weight PAHs with four or more rings. With some exceptions (Shuttleworth and Cerniglia, 1996; Ho et al., 2000; Kim et al., 2005; Lai et al., 2012), high molecular weight PAHs are cometabolized with the low molecular PAHs, salicylate, and other plant-derived compounds being the primary substrates (van Herwijnen et al., 2003; Rentz et al., 2005, 2008; Peng et al., 2008).

In the initial steps of aerobic degradation, molecular oxygen is required to oxidize and cleave the aromatic ring (Cerniglia, 1992). In the absence of oxygen (anaerobic conditions), the degradation rates of PAHs are much lower (Eriksson et al., 2003). Due to the usually limited amount of air in many environments, oxygen is delivered through aeration when biodegradation needs to be accelerated (Lu et al., 2011). When assessing biodegradation potential, the presence of populations needs to be verified which are able to aerobically degrade, and ideally mineralize, the substrates. Stable isotope probing (SIP) is one of molecular methods for determining biodegradation potential (Chen and Murrell, 2010; Chen et al., 2010; Uhlik et al., 2013a). Using this method, many important findings regarding PAH-degrading organisms have been obtained, such as the discovery of a previously undescribed naphthalene-utilizing bacterium *Polaromonas naphthalenivorans* (Jeon et al., 2003, 2004), confirmation of salicylate as an inducer of naphthalene catabolism (Singleton et al., 2005), and detection of *in situ* PAH-degrading populations (Padmanabhan et al., 2003). Recently, SIP has been combined with high-throughput sequencing to gain a more in-depth insight into PAH-utilizing microbial populations (Singleton et al., 2011).

In addition to oxygen, temperature is an important factor in PAH biodegradation. The ambient temperatures typically used for the majority of cultivation-based studies often inadequately represent temperatures found in many environments. Although, various studies show that limited degradation is associated with lower temperatures (Eriksson et al., 2003; Brakstad et al., 2008; Okere et al., 2012; Leewis et al., 2013), psychrotolerant PAH-degrading organisms have also been found (Ahn et al., 2005; Sørensen et al., 2010).

In this study, we investigated microbial populations responsible for the biodegradation of PAHs in contaminated sediment after aeration. We hypothesized that (i) contaminated sediment contains populations with a potential to aerobically degrade naphthalene and other PAHs; (ii) aeration will result in metabolic activity in these populations; and (iii) aerobic biodegradation will occur at 10°C—a temperature more similar to that found *in situ*. SIP was coupled with the sequencing of 16S rRNA gene pyrotags to identify naphthalene-utilizing populations. The results of the study were confirmed by culture-based experiments and isolation of active naphthalene degraders in pure cultures.

## Materials and methods

### Sediment samples

Sediment samples were collected in November 2011 from a lake in Romania located near an oil refinery, where aeration is used to stimulate intrinsic bioremediation. Sediment dry weight (determined at 105°C) was 16% and pH was 8.0 ± 0.5, with PAH content being listed in Table 1. Immediately after transport to the laboratory, the samples were used for the construction of SIP microcosms and cultivation-based studies.

**Table 1:** **The content of PAHs in the analyzed sediment**.

**Polyaromatic hydrocarbon**	**Content (mg/kg)**
Naphthalene	1160
Acenaphthylene	27.7
Acenaphthene	581
Fluorene	779
Phenanthrene	2040
Anthracene	293
Fluoranthene	415
Pyrene	391
Benz[*a*]anthracene	122
Chrysene	88.5
Benzo[*b*]fluoranthene	75.1
Benzo[*k*]fluoranthene	30.4
Benzo[*a*]pyrene	64.0
Indeno[1,2,3-*cd*]pyrene	13.8
Benzo[*ghi*]perylene	13.8
Dibenz[*ah*]anthracene	6.90
Sum of 16 PAHs	6100

*Standard error is ±30%*.

### SIP microcosms

Duplicate SIP microcosms were constructed in 250 mL sterile flasks with 0.5 mg of uniformly ^13^C_10_-labeled naphthalene (>99% ^13^C, Sigma-Aldrich, USA), which was applied as a solution in acetone (concentration 0.05 g/ml). After the acetone was allowed to evaporate leaving behind naphthalene crystals, 2.5 g of settled sediment was added with basal mineral salt solution (Uhlik et al., 2009) to obtain a final volume of 25 mL. The flasks were sealed, incubated at 10 and 20°C for 4, 7, and 14 days with shaking at 130 rpm. Parallel incubation with unlabeled naphthalene (Sigma-Aldrich, USA) was carried out for 14 days. After the incubation time had passed, the microcosms were stored at −20°C prior to downstream processing.

### Isotopic composition of headspace CO_2_

Microcosms were thawed at laboratory temperature and then kept in warm water for 20 min to release dissolved gas. Using a Hamilton GASTIGHT syringe, 250 μL of the headspace gas was transferred to a 12 mL W938 borosilicate vacutainer (IVA Analysentechnik e.K., Germany) that had been flush-filled with helium. The isotopic composition of headspace CO_2_ was analyzed using a Delta V Advantage IRMS (Thermo Fisher Scientific, USA) coupled with a Gasbench II device equipped with a single cryotrap.

### Isolation of labeled DNA

Sediment suspensions from SIP microcosms and from initial time zero (*T*_0_) soil samples were pelleted at 5000 × g for 10 min and frozen until further processing. The metagenomic DNA was isolated using PowerMax™ Soil DNA Isolation Kits (MoBio Laboratories Inc., USA) and concentrated by ethanol precipitation with glycogen (Roche, Switzerland). DNA concentration in all samples was normalized to 100 ng/μL by diluting with PCR grade water (Sigma-Aldrich, USA). Subsequently, 8 μL of DNA solution was mixed with cesium trifluoroacetate (Amersham, UK) pre-diluted to a concentration of 1.6 g/mL in a 2 mL ultracentrifugation cuvette. Labeled DNA was isolated by isopycnic centrifugation performed on a Discovery 90 Ultracentrifuge using a TFT-80.2 Fixed-Angle Ultraspeed Centrifuge Rotor (Sorvall, USA) under conditions of 145,000 × *g* for >70 h. Each gradient was fractionated into 60 μL fractions using a Beckman Fraction Recovery System (Beckman Coulter, USA) and a Harvard Pump 11 Plus Single Syringe (Harvard Apparatus, USA) by replacement with water at a flow rate of 200 μL/min.

Isopycnic centrifugation and gradient fractionation of the samples were run in parallel with two blanks (DNA replaced with water) which were used to ensure reproducibility and accuracy of both centrifugation and fractionation by measuring the buoyant density of each fraction with a Digital Handheld Refractometer (Reichert Analytical Instruments, USA). DNA was retrieved from cesium trifluoroacetate by isopropanol precipitation with glycogen (Uhlik et al., 2009).

The distribution of DNA as a function of buoyant density was determined by qPCR with primers 786f, 5′-GATTAGATACCCTGGTAG-3′ and 939r, 5′-CTTGTGCGGGC CCCCGTCAATTC-3′ targeting the 16S rRNA gene. DNA quantification was performed in 12 μL reactions using a DyNAmo Flash SYBR Green qPCR Kit (Thermo Scientific, USA), 5 pmol of each primer, and 2 μL of DNA. Cycling was performed in a CFX96 Real-Time System (Bio-Rad, USA) following the cycling program of a denaturation at 95°C for 7 min, followed by 35 cycles at 95°C for 20 s, 55°C for 30 s, and 72°C for 30 s, followed by the final extension at 72°C for 2 min. Fractions with ^13^C-enriched DNA were combined and further analyzed as ^13^C-DNA. Corresponding fractions from the gradients with unlabeled DNA and *T*_0_ DNA were combined and analyzed as controls.

### Pyrosequencing

Amplicons of 16S rRNA genes were generated using a two-step PCR approach which was adapted and modified from Berry et al. (2011). The first PCR was performed with primers 515f, 5′-GTGCCAGCMGCNGCGG-3′ and 1406r, 5′-ACGGGCGGTGWGTRC-3′ in 20 μL reactions with 0.2 mM dNTPs (Thermo Scientific, USA), 0.5 μM primers, 0.1 mg/mL of BSA, and 0.4 U of Phusion Hot Start II DNA Polymerase with the corresponding HF buffer (Thermo Scientific, USA). Thermocycling conditions were set as follows: 98°C for 3 min, followed by 35 cycles of 98°C for 10 s, 61°C for 30 s, and 72°C for 30s, with the final elongation at 72°C for 10 min. The resulting PCR products were used as templates for a second PCR run, in which the primers 515f and 1406r were fused with sequencing adapters (454 Sequencing Application Brief No. 001-2009, Roche) at 5′-ends. The forward primers contained different tags (454 Sequencing Technical Bulletin No. 005-2009, Roche) which allowed for multiplexing. PCR was performed in 25 μL reactions with 0.5 μL of the template, 0.2 mM dNTPs (Thermo Scientific, USA), 1 μM primers, 0.1 mg/mL of BSA, and 0.5 U of Phusion Hot Start II DNA Polymerase with the corresponding HF buffer (Thermo Scientific, USA). The cycling program was set to 98°C for 3 min, followed by 10 cycles at 98°C for 10 s, 61°C for 30 s, and 72°C for 30 s, with the final elongation at 72°C for 10 min. Finally, the PCR products were pooled and purified using AMPure XP Beads (Agencourt, Beckman Coulter, USA) to remove residual primer-dimers according to the manufacturer's instructions. Amplicons were unidirectionally sequenced from the forward primer using GS FLX+ chemistry followed by standard analysis of signal processing (Roche).

### Sequence analysis

Pyrosequencing data were processed with the aid of the mothur software package, version 1.31.1 (Schloss et al., 2009). The flowgrams, trimmed to 650–800 flows, with the number of differences in barcode and primer being set to 0, were denoised until the change in flowgram correction reached 10^−6^. Barcode and primer sequences were removed from the fasta sequences generated, and unique sequences were aligned against merged SILVA bacterial and archaeal reference alignments. Sequences that did not align well or were shorter than 400 bp were removed, and unique sequences were pre-clustered using the pseudo-single linkage algorithm merging sequences with a difference of 1 bp per 100 bp of sequence length. Chimeric sequences were identified using the Perseus algorithm (Quince et al., 2011) and removed from the data set. Classification of valid sequences was performed against Ribosomal Database Project (RDP) (Cole et al., 2009; Cole and Tiedje, 2014) reference files (trainset 9). Singletons and contaminating sequences (mitochondria, chloroplasts, eukaryotic or unknown sequences) were removed from the data set. Final sequences were clustered by average linkage algorithm at 3% to create operational taxonomic units (OTUs). The most abundant sequence in each OTU was selected as a representative sequence and was submitted to RDP Seqmatch (Cole et al., 2009) in order to identify the closest type strains. The pyrosequencing error rate was determined by analyzing mock community sequences as described previously (Uhlik et al., 2013b).

### Bacterial extraction and cultivation

The sediment samples were cultivated in liquid enrichment cultures (Kurzawova et al., 2012), with naphthalene as the sole carbon source. Sediment suspensions were prepared in liquid mineral salt solution (10%, w/v, Uhlik et al., 2009) with the addition of naphthalene crystals (~1 mg/mL) and transferred at 1 week intervals for 8 weeks at 10 and 20°C. Finally, 100 μL of each suspension were diluted to extinction in 0.85% (w/v) NaCl solution and plated on solid mineral media (Uhlik et al., 2011) with crystalline naphthalene in the lid (~10 mg). After colonies appeared, they were re-inoculated in a liquid mineral salt solution and incubated with naphthalene to confirm the growth on this substrate. At the same time, each of the colonies was re-inoculated on a Plate Count Agar (PCA), and after 48 h of growth at 28°C the cultures were identified using an Autoflex Speed MALDI-TOF/TOF mass spectrometer and MALDI Biotyper 3.1 software (Bruker Daltonik, Germany) equipped with 5627 MSP library (released in December 2013, Bruker Daltonics, Germany) in a manner similar to that described elsewhere (Uhlik et al., 2011). In order to match the sequences from pyrosequencing analysis with isolated cultured representatives, 16S rRNA genes were sequenced for isolates utilizing naphthalene and those that were not identified by MALDI TOF MS. Briefly, the genomic DNA was isolated using thermal lysis, in which a PCA-grown colony was transferred to a microtube, mixed with 50 μL of PCR grade water (Sigma-Aldrich, USA) and incubated at 95°C for 20 min. Afterwards, the cell debris was pelleted, and the supernatant containing the genomic DNA was used for PCR. The 16S rRNA genes were amplified with primers 8f (Suzuki and Giovannoni, 1996) and 1406r (described above) in 20 μL reactions with 0.2 mM dNTPs (Thermo Scientific, USA), 0.2 μM primers (Generi Biotech, Czech Republic), 0.1 mg/mL of BSA, and 0.02 U/μL of iProof™ DNA polymerase with the corresponding buffer (Bio-rad, USA). The cycling program was set to 98°C for 3 min, followed by 25 cycles at 98°C for 10 s, 56°C for 20 s, and 72°C for 40 s, with the final elongation at 72°C for 10 min. The PCR products were re-amplified in a reconditioning step (Thompson et al., 2002), in which 5 μL of PCR product was used as a template in another six-cycle PCR with the total volume of 50 μL with the same primers and other chemicals in the same concentrations as were used for the first PCR. The final PCR products were purified using AMPure XP Beads (Agencourt, Beckman Coulter, USA) following the manufacturer's instructions. Unidirectional sequencing from the forward primer was performed at the Sequencing Laboratory of the Faculty of Science, Charles University in Prague, Czech Republic. The 16S rRNA gene sequences in bacterial isolates and pyrosequencing libraries were compared by local BLAST using a BLAST+ Release 2.2.26 (Zhang et al., 2000).

### Dioxygenase gene detection

Primers to detect naphthalene dioxygenase large subunit genes were designed on the basis of sequences obtained from a *npah* gene set in the Functional Gene Pipeline and Repository (Fish et al., 2013). Only sequences longer than 1200 bp and with a cutoff score >900 were used. Primers were designed manually in conserved regions of the gene after aligning the sequences using MEGA5 (Tamura et al., 2011). The sequences of the primers were as follows: 301f, 5′-TGCRRYTAYCAYGGCTGG-3′ and 1099r, 5′-CCATRTTSTCRKTRTCKTC-3′. The region to be amplified begins and ends at nucleotide positions 301 and 1099, respectively, of *Pseudomonas putida* NCIB 9816-4 *nahAc*, yielding an expected amplicon size of ~799 bp. The forward primer binding site flanks the Rieske center. *In silico* testing of the primer set using the *Probe Match Search* tool in FunGene (Fish et al., 2013) showed that the primers target: *Pseudomonas*-like *nahAc, ndoAc, doxAc, pahAc* genes (Kurkela et al., 1988; Denome et al., 1993; Simon et al., 1993; Takizawa et al., 1994; Chauhan et al., 2011); *Comamonas*-like *pahAc* genes (Moser and Stahl, 2001); *Ralstonia* or *Polaromonas*-like *nagAc* genes (Fuenmayor et al., 1998; Jeon et al., 2006); and *Burkholderia, Acidovorax*, or *Alcaligenes*-like *phnAc* genes (Laurie and Lloyd-Jones, 1999).

PCR amplification was performed in 20 μL reactions with 0.2 mM dNTPs (Thermo Scientific, USA), 0.8 μM primers, 0.1 mg/mL of BSA, and 0.02 U/μL of Phusion Hot Start II DNA Polymerase with the corresponding HF buffer (Thermo Scientific, USA). The cycling program was set to 98°C for 3 min, followed by 35 cycles at 98°C for 25 s, 56°C for 20 s, and 72°C for 30 s, with the final elongation at 72°C for 10 min. PCR products were processed in a way similar to that described for 16S rRNA genes of the isolated bacteria. The sequences obtained were translated into proteins and aligned with known sequences using MEGA5 (Tamura et al., 2011). The tree was constructed using the Maximum Likelihood method.

### Degradation of PAHs

The growth curves of isolated strains were assessed in liquid mineral salt solution, with naphthalene as the sole carbon source at both 10 and 20°C. The cells were pre-grown in liquid mineral salt solution with naphthalene. After a late exponential phase had been achieved, the cultures were filtered through sterile cotton wool in order to remove remaining naphthalene crystals, centrifuged (10 min; 5000 × g) and washed twice with fresh mineral salt solution. Finally, the cells were resuspended in a small amount of mineral salt solution. The degradation assay was performed in triplicate in 8 mL glass vials with screw caps lined with polytetrafluoroethylene. Resuspended cells were diluted with mineral salt solution to the optical density OD_600nm_ of 0.3 and final volume of 4 mL. The PAHs were spiked in the empty vial from PAH stock solutions in hexane which was allowed to evaporate in the fume hood before the cells were added. The final concentration of each PAH (naphthalene, acenaphthene, fluorene, phenanthrene, anthracene, fluoranthene, and pyrene, all from Sigma-Aldrich, USA) in the final mixture was 20 mg/L. After 4, 7, and 14 days of incubation (10 or 20°C; orbitally shaken at 130 rpm) in the dark, remaining PAHs were extracted twice with 1 mL of hexane and 150 mg/L benzene (internal standard) for 2 h (28°C; orbitally shaken at 130 rpm), eventually combining both extracts. Each assay was performed in triplicate. PAHs in the hexane fraction (10 μL injection) were quantified using HPLC (HP1100) analysis with a 150 × 2 mm × 5 μm Luna C18(2) column (Phenomenex) and isocratic elution with 35% acetonitrile:65% water with flow rate 0.8 mL/min. PAHs were detected spectrophotometrically (benzene 202 nm, naphthalene 220 nm, acenaphthene 226 nm with reference 276 nm for fluorene correction, pyrene 240 nm with reference 287 nm for fluoranthene correction, fluorene 263 with reference 308 nm for acenaphthene correction, fluoranthene 286 with reference 345 nm for pyrene correction, phenanthrene and anthracene 250 nm), the retention times were as follows: benzene 4.1 min, naphthalene 12.0 min, fluorene 28.6 min, acenaphthene 26.8 min, phenanthrene 36.1 min, anthracene 42.6 min, fluoranthene 58.6 min, and pyrene 61.7 min. ChemStation software was used for HPLC measurement, integration of the chromatograms as well as quantification of PAHs.

Degradation efficiency was evaluated using the Welch Two Sample *t*-test in R (R Development Core Team, 2009) at a 5% significance level.

### Sequence deposition

Pyrosequencing reads were deposited in NCBI Sequence Read Archive under accession number SRP063975. 16S rRNA gene and PAH dioxygenase gene sequences were deposited in the GenBank database under accession no. KJ643424–KJ643427 and KJ643430–KJ643433, respectively.

## Results

### SIP: Headspace ^13^CO_2_

The isotopic composition of headspace CO_2_ was monitored in order to assess the mineralization of ^13^C-naphthalene. The results showed that mineralization of ^13^C-naphthalene was faster at 20 than 10°C (Table 2). The amount of evolved ^13^CO_2_ did not increase significantly between day 7 and 14 at either temperature and indicated that between 50 and 55% of the original ^13^C-naphthalene was respired.

**Table 2:** **Headspace CO_2_ analysis and the amount of ^13^CO_2_ evolved**.

**Parameter**	**10**^**°**^**C**			**20**^**°**^**C**		
	**Day 04**	**Day 07**	**Day 14**	**Day 04**	**Day 07**	**Day 14**
Headspace CO_2_ concentration (%)	0.95 ± 0.03	4.27 ± 0.22	5.62 ± 1.00	3.60 ± 0.31	4.66 ± 0.05	5.56 ± 0.14
^13^CO_2_ evolved (nmol)	987 ± 137	21,647 ± 1361	20,785 ± 947	18,734 ± 1803	19,555 ± 516	20,476 ± 18
^13^C-naphthalene mineralized (%)^a^	2.5 ± 0.4	55 ± 3	53 ± 2	48 ± 5	50 ± 1	52 ± 0

*The measurements were taken on independent biological duplicates*.

a *The amount is based on chemical equation of naphthalene mineralization: C_10_H_8_ + 12O_2_ → 10CO_2_ + 4H_2_O*.

### Quantitative PCR and “heavy” DNA isolation

**Figure 1:**
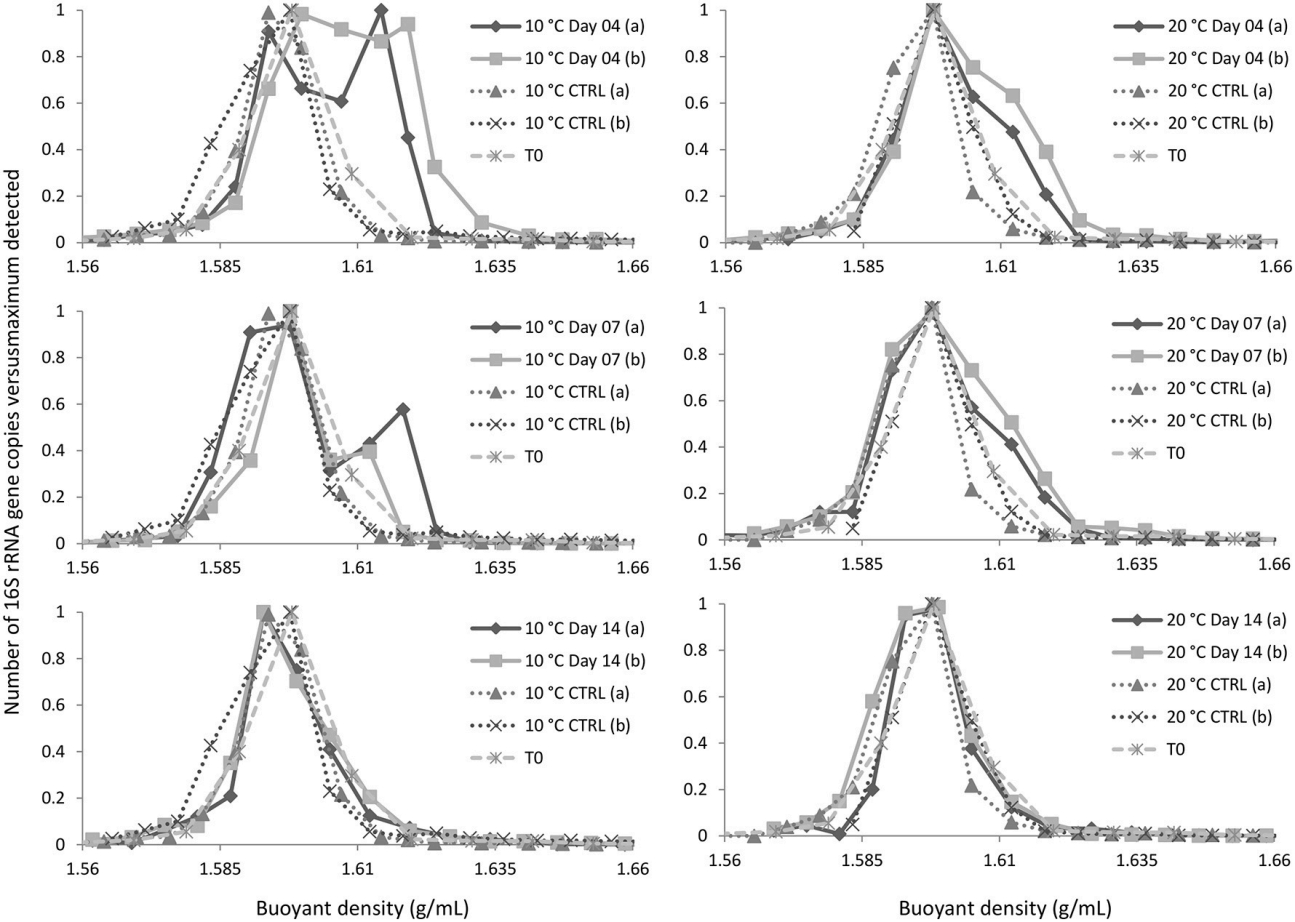
**Distribution of DNA in the gradient**. CTRL and *T*_0_ refer to, respectively, DNA isolated from parallel incubations with unlabeled naphthalene and initial sediment sample. Symbols (*a*) and (*b*) refer to replicate experimental data.

Q-PCR analyses of gradient fractions were performed to examine the distribution of DNA throughout the gradient and to localize ^13^C-DNA-containing fractions (Figure 1). The dominant peak was recorded at buoyant densities (BDs) of 1.59–1.60 g/mL and represented unlabeled DNA. While a substantial amount of ^13^C-enriched (or “heavy”) DNA was recorded at BDs >1.62 g/mL, only minor quantities of DNA were detected at the same BDs in control samples (fractionated DNA from parallel incubations with unlabeled naphthalene or from initial (*T*_0_) sediment samples). The fractions with BDs >1.62 g/mL were combined and further analyzed as “heavy” DNA along with the corresponding fractions from *T*_0_ (control) samples.

### 16S rRNA gene profiling

Analysis of pyrosequencing reads of the mock community according to Schloss et al. (2011) showed that the sequence error was 10^−3^. The total number of valid sequence reads was 80,160 with an average number of 4715 reads per sample/replicate, including controls. Prior to further analyses the sizes of the libraries were normalized according to Schloss et al. (2011) to the number of reads in the least library, which was 1939.

**Figure 2:**
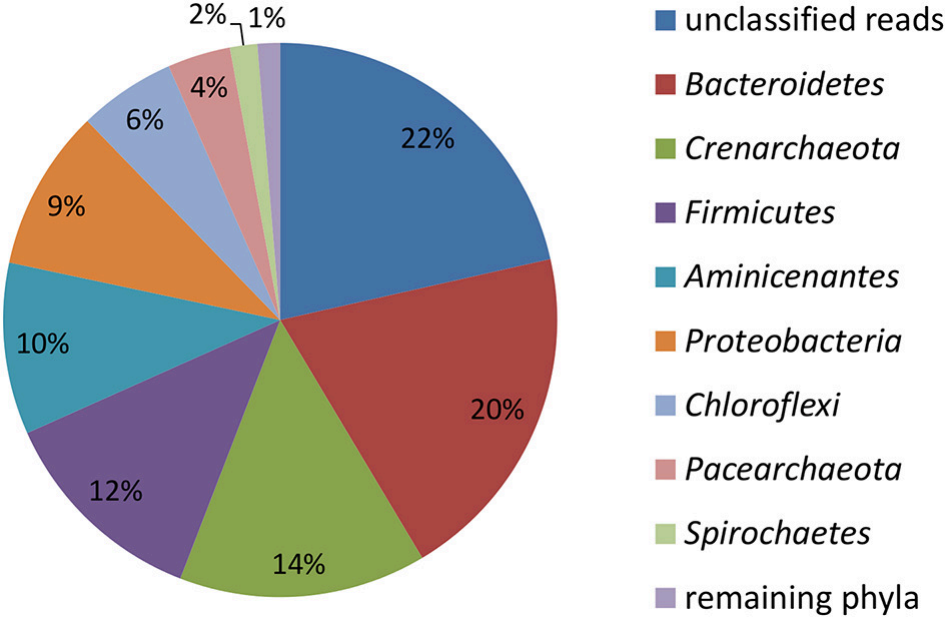
**Relative abundance of reads affiliated with phyla in the initial total community DNA isolated from the sediment prior to SIP incubations calculated from the total number of 4024 reads**.

16S rRNA gene profiling indicated that only members of *Proteobacteria* derived carbon from naphthalene (Table 3). In addition to the clearly predominant pseudomonads at both temperatures, naphthalene-derived ^13^C was detected in *Stenotrophomonas, Acidovorax*, and *Comamonas*, and sporadically in other *Proteobacteria*. The dominant pseudomonads comprised three OTUs, of which the most abundant OTU clustered with the *Pseudomonas fluorescens* group (with closest type strains being *Pseudomonas veronii* CIP 104663, *Pseudomonas proteolytica* CMS 64, *Pseudomonas brenneri* CFML 97-391T, and *Pseudomonas gessardii* CIP 105469) followed by the *Pseudomonas syringae* group (namely *Pseudomonas cichorii* LMG 2162T) and *Pseudomonas peli* R-20805. At 10°C, the number of *Pseudomonas* sequences in libraries from both 4- and 7-day incubation exceeded 99%, indicating a dominant role for this taxon in the primary consumption of naphthalene. *Stenotrophomonas, Acidovorax*, and *Comamonas* were detected only after 14 days of incubation. At 20°C, these sequences appeared earlier over the course of incubation. Other sequences also appeared in the 14-day incubation library at lower abundance rates (Table 3). Most of the sequences detected in SIP were not detected in the initial total community DNA, although more than 99% coverage was achieved according to Schloss et al. (2011). The composition of the initial community DNA is shown in Figure 2.

**Table 3:** **Bacterial populations deriving carbon from naphthalene, either directly or via cross-feeding, as detected at different time points at 10 and 20°C**.

**Phylogenetic affiliations^a^**	**Closest RDP SeqMatch type strain**	**Similarity score^b^**	**Accession no. of the type strain**	**10**^**°**^**C**						**20**^**°**^**C**						**% in TC^c^**
				**Day 04**		**Day 07**		**Day 14**		**Day 04**		**Day 07**		**Day 14**		
***GAMMAPROTEOBACTERIA***																
***Pseudomonadaceae***																
*Pseudomonas*	*P. veronii* CIP 104663, *P. brenneri* CFML 97-391, *P. gessardii* CIP 105469, *P. proteolytica* CMS 64	1.000	AF064460, AF074384, AJ537603, AF268968	>99%	>99%	>99%	>99%	94%	97%	94%	85%	91%	83%	64%	66%	ND
*Pseudomonas*	*P. cichorii* LMG 2162	1.000	Z76658	< 0.5%	< 0.5%	< 0.5%	< 0.5%	< 0.5%	< 0.5%	6%	9%	6%	7%	3%	7%	ND
*Pseudomonas*	*P. peli* R-20805	0.993	AM114534	ND	ND	ND	< 0.5%	2%	1%	< 0.5%	< 0.5%	< 0.5%	1%	2%	1%	ND
***Xanthomonadaceae***																
*Stenotrophomonas*	*S. rhizophila* e-p10	1.000	AJ293463	ND	ND	ND	ND	2%	1%	< 0.5%	2%	1%	4%	14%	13%	ND
*Pseudoxanthomonas*	*P. spadix* IMMIB AFH-5	0.998	AM418384	ND	ND	ND	ND	ND	ND	ND	ND	ND	ND	< 0.5%	1%	ND
***Shewanellaceae***																
*Shewanella*	*Shewanella* spp.	1.000	Multiple hits	ND	ND	ND	ND	< 0.5%	< 0.5%	ND	< 0.5%	ND	ND	< 0.5%	< 0.5%	ND
***BETAPROTEOBACTERIA***																
***Comamonadaceae***																
*Acidovorax*	*A. defluvii* BSB411	1.000	Y18616	ND	ND	ND	ND	< 0.5%	< 0.5%	< 0.5%	1%	1%	2%	6%	4%	ND
*Comamonas*	*C. testosteroni* ATCC 11996	0.998	M11224	ND	ND	ND	ND	< 0.5%	< 0.5%	< 0.5%	1%	< 0.5%	1%	5%	3%	ND
*Hydrogenophaga*	*H. atypica* BSB 41.8, *H. defluvii* BSB 9.5	0.991	AJ585992.3	ND	ND	ND	ND	ND	ND	ND	ND	ND	ND	< 0.5%	< 0.5%	0.05%
***Rhodocyclaceae***																
*Georgfuchsia*	*Sulfuritalea hydrogenivorans* DSM 22779	0.967	AB552842	ND	ND	ND	ND	ND	ND	ND	ND	< 0.5%	1%	2%	< 0.5%	0.05%
***Hydrogenophilaceae***																
*Thiobacillus*	*T. thioparus* ATCC 8158	0.993	M79426	ND	ND	ND	ND	ND	ND	ND	ND	ND	ND	1%	1%	0.2%
***Alcaligenaceae***																
*Bordetella*	*B. trematum* DSM 11334	0.998	AJ277798	ND	ND	ND	ND	ND	ND	ND	ND	ND	ND	< 0.5%	< 0.5%	ND
***ALPHAPROTEOBACTERIA***																
***Caulobacteraceae***																
*Brevundimonas*	*B. bullata* IAM 13153	1.000	D12785	ND	ND	ND	ND	ND	ND	ND	ND	ND	ND	< 0.5%	< 0.5%	ND

*The values shown are relative abundances of reads per library*.

*ND, not detected*.

a *Phylogenetic affiliations are based on Ribosomal Database Project (RDP) Classifier*.

b *Similarity reports the percent sequence identity over all pairwise comparable positions*.

c *Percent of reads in initial total community DNA*.

### SIP vs. cultivation-based method

Aerobic naphthalene-utilizing bacteria were enriched in cultures, with naphthalene as the sole carbon source. Dilution to extinction and selective plating on mineral media with naphthalene resulted in the isolation of seven bacterial cultures, four of which were proved to utilize naphthalene as a sole carbon and energy source (Table 4). These naphthalene-utilizing cultures were identified as *P. veronii, P. gessardii, Pseudomonas* sp., and *Comamonas testosteroni*.

**Table 4:** **Naphthalene-utilizing bacterial isolates extracted from the sediment and their identification based on MALDI-TOF MS with MALDI Biotyper and/or 16S rRNA gene analysis**.

**Isolate designation**	**MALDI Biotyper-based identification**	**Score^a^**	**Closest RDP SeqMatch type strain; similarity score^b^**	**Accession no. of the type strain**	**GenBank accession no**.	
					**16S rRNA**	***npah*^c^**
20a2	*Pseudomonas veronii*	+++	*Pseudomonas veronii* CIP 104663; 1.000	AF064460	KJ643424	KJ643430
10a3	*Ps. gessardii*	++	*Pseudomonas gessardii* CIP 105469; 1.000	AF074384	KJ643425	KJ643431
20b3	No reliable identificaiton	–	*Pseudomonas taiwanensis* BCRC 17751; 0.992	EU103629	KJ643426	KJ643432
20a3	*Comamonas testosteroni*	+++	*Comamonas testosteroni* ATCC 11996; 0.998	M11224	KJ643427	KJ643433

*ND, not detected*.

a *Scores: +++, highly probable species identification; ++, probable species identification; +, probable genus identification; –, no reliable identification*.

b *Similarity reports the percent sequence identity over all pairwise comparable positions*.

c *Naphthalene dioxygenase gene*.

Furthermore, the culture-based study permitted a more reliable classification of the pyrosequencing reads to a species level. As shown by local BLAST alignment, all the 16S rRNA genes of the sequenced isolates shared >99% identity with the most abundant reads in ^13^C-DNA pyrosequencing libraries, with differences detected only in homopolymeric regions located toward the distal ends of the pyrosequencing reads. These were very likely to be technology-based errors as pyrosequencing is mainly affected by errors in homopolymeric regions (Kunin et al., 2010). Such errors tend to accumulate especially toward the distal end of the reads (Schloss, 2013). The local BLAST alignment-based comparison of 16S rRNA gene sequences from the pure cultures and the pyrosequencing libraries indicated that the most abundant OTU affiliated to *Pseudomonas* (Table 3) was associated with two taxa from the *P. fluorescens* group, namely *P. veronii* and *P. gessardii* (isolates 20a2 and 10a3, respectively; Table 4). Both of these isolates differed from the most abundant OTU's representative sequence only at one position in a homopolymeric region. The differences in the *P. veronii* and *P. gessardii* 16S rRNA genes were located outside the pyrosequenced region, resulting in their inclusion in a common OTU. The OTU described by the representative of *P. cichorii* (Table 3) seemed to be represented rather by *P. taiwanensis* from the *P. putida* group (isolate 20b3; Table 4); however, as the findings were not confirmed by mass spectrometry, species-level identification was inconclusive. The OTU identified as *Comamonas* was confirmed to be associated with *C. testosteroni*.

### Naphthalene dioxygenases

**Figure 3:**
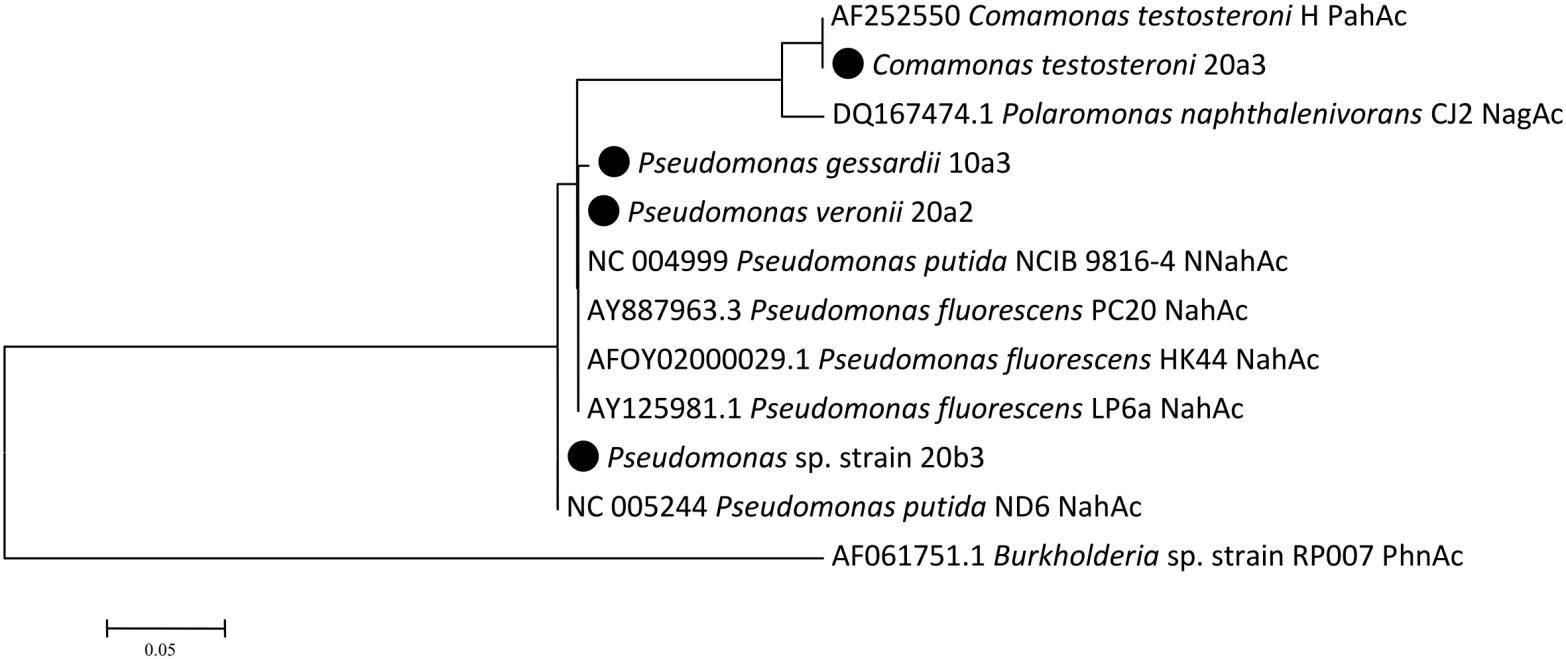
**Naphthalene dioxygenase large subunit Maximum Likelihood tree**. There were a total of 241 positions in the final dataset. Isolates from this study are highlighted with the black circle; public sequences are shown with their GenBank accession numbers.

The detection of naphthalene dioxygenase large subunit genes (*nahAc*) was performed with newly designed primers reported in the Materials and Methods Section. Naphthalene dioxygenase genes were detected in all strains that utilized naphthalene in the culture-based experiments. Their sequences clustered with sequences previously detected in pseudomonads and comamonads (Figure 3). The genes in isolates 20a2 and 10a3 differed only in one nucleotide, resulting in one amino acid difference (corresponding to position 219 of *Pseudomonas putida* NCIB 9816-4 NahAc).

### Degradation assay

**Figure 4:**
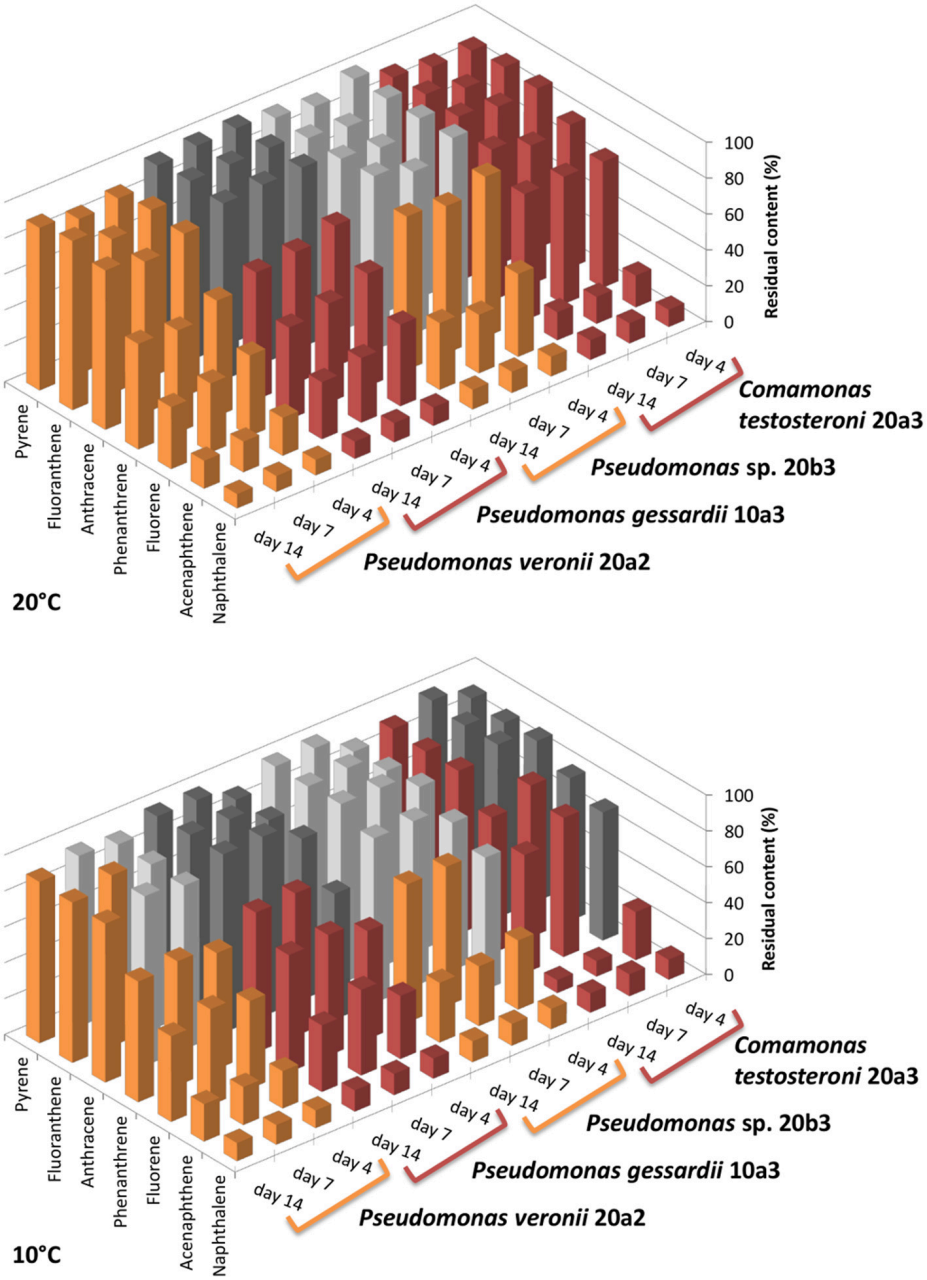
**Residual content of selected PAHs in medium after the degradation experiment with isolated bacteria**. The degradation test was performed at 10 and 20°C. Initial concentration of each PAH was 20 mg/L (~100%, day 0), the experiment was terminated on day 4, 7, and 14. The results shown are mean values of three biological replicates, the colors used for different isolates (orange or red) are equal in meaning and indicate statistical significance of the degradation as determined by Welch Two Sample *t*-test (α = 0.05).

The degradation assay focused on the seven most abundant PAHs in the sediment, namely naphthalene, acenaphthene, fluorene, phenanthrene, anthracene, fluoranthene, and pyrene. The results of the degradation assay (Figure 4) showed that the highest degradation capabilities could be ascribed to *P. veronii* 20a2 and *C. testosteroni* 20a3. These strains were able to degrade all the PAHs tested, including anthracene and the four-ring PAHs fluoranthene and pyrene. Degradation efficiency was higher at 20°C where statistically significant depletion of all PAHs was detected after just 4 days. Naphthalene and acenaphthene were efficiently degraded by all strains at both temperatures as early as on day 4.

## Discussion

Studying microbial diversity in contaminated environments is essential for predicting the bioremediation potential of autochthonous populations. In the context of bioremediation, the flow of carbon from contaminants through the microbial community is an important issue; detecting both primary degraders and cross-feeding populations enables all microorganisms essential to the completion of the degradation process to be identified (Uhlik et al., 2013a). SIP combined with time-course sampling is one such technique which allows for the detection of members of the community through which the labeled carbon flows.

### Fate of naphthalene-derived carbon

When employing SIP, it is important not to exceed the environmentally relevant concentrations of the labeled substrate (Neufeld et al., 2007); in this study, the amount of ^13^C-naphthalene used was 0.5 mg per 2.5 g of the sediment, less than five times the naphthalene concentration measured in the sediment (Table 1). Unsurprisingly, the amounts of ^13^CO_2_ evolved by ^13^C-naphthalene respiration in this study indicated a faster metabolism at 20 vs. 10°C (Table 2). The diversity of populations incorporating the label from ^13^C-naphthalene did not differ significantly at the two temperatures tested, with only the carbon flow at 10°C being slower.

Pseudomonads dominated the acquisition of naphthalene-derived carbon (Table 3). They were the only taxon detected which contained the label on days 4 and 7 at 10°C and were also dominant at 20°C. The detection of pseudomonads as naphthalene-utilizing bacteria was not surprising, as many studies have described members of this genus to be involved in PAH utilization (Kuiper et al., 2001; Samanta et al., 2002; Singleton et al., 2005, 2011; Jones et al., 2011; Lu et al., 2011; Uhlik et al., 2012), some of which were members of the *P. fluorescens* group (Whitman et al., 1998; di Gennaro et al., 2006; Bučková et al., 2013). Our study reports an overwhelming dominance of one OTU (Table 3) affiliated with the *P. fluorescens* group during the degradation of naphthalene. The culture based experiments further determined that this OTU spanned two closely related species of pseudomonads, namely *P. veronii* and *P. gessardii* (Table 4). The degradation assay highlighted the exceptional degradation capabilities of the *P. veronii* culture (Figure 4); in addition to the virtual complete degradation of naphthalene, all other PAHs, including those with four-rings, were transformed by this culture at both 10 and 20°C. The naphthalene dioxygenase sequence detected in *P. veronii* was identical to that of *P. putida* NCIB 9816-4 (Figure 3). This enzyme has been reported to oxidize phenanthrene, anthracene (Jerina et al., 1976), acenaphthene (Selifonov et al., 1996) and fluorene (Resnick and Gibson, 1996) in addition to naphthalene.

Although the observed degradation capacity of *P. veronii* might thus have resulted from the activity of this enzyme, the presence of other dioxygenases in the isolate cannot be ruled out. It has previously been reported that *Pseudomonas rhodesiae*, which is closely related to *P. veronii* and *P. gessardii*, contains redundant dioxygenases responsible for PAH degradation (Kahng et al., 2002). Such a scenario is quite plausible given that the naphthalene dioxygenase genes of *P. veronii* and *P. gessardii* differed by only one amino acid although the degradation capabilities of the strains differed markedly (Figure 4). However, resolving these issues is beyond the scope of this study and remains to be elucidated in the future. Indeed, interactions among PAHs during their degradation have previously been reported to be highly complex, ranging from cometabolism (Cerniglia, 1992; Shuttleworth and Cerniglia, 1995; Habe and Omori, 2003; Rentz et al., 2005; Peng et al., 2008) via competition (Stringfellow and Aitken, 1995) to inhibition (Bouchez et al., 1995; Shuttleworth and Cerniglia, 1996).

*C. testosteroni* (Tables 3, 4), the other population deriving carbon directly from naphthalene, has also been previously described as a PAH-degrading species utilizing naphthalene, phenanthrene or anthracene as its carbon sources (Goyal and Zylstra, 1996, 1997). Additionally, the naphthalene dioxygenase large subunit from *C. testosteroni* clustered with that of previously described comamonads (Figure 3).

### Primary consumers vs. cross-feeders

Through the combination of SIP and culture-based methods, we were able to observe that some populations directly involved in ^13^C-naphthalene degradation were likely to have utilized labeled intermediate metabolites of naphthalene rather than naphthalene itself. In fact, *Stenotrophomonas* sp., *Stenotrophomonas maltophilia*, and *Acidovorax defluvii* were among the bacteria isolated from liquid enrichment cultures which were unable to utilize naphthalene as the sole carbon source when their colonies were selected and reinoculated into liquid medium. It has previously been reported that such a secondary screening of colonies is required as some may grow on suspension carryover (Leigh et al., 2002). In addition, screening of *Stenotrophomonas* and *Acidovorax* isolates for the presence of naphthalene dioxygenase genes did not prove the presence of the dioxygenase genes studied. Stenotrophomonads are commonly associated with the utilization of aromatics, including those that are chlorinated (Padmanabhan et al., 2003; Gentry et al., 2004; Leigh et al., 2007; Koubek et al., 2013; Uhlik et al., 2013b), which are mineralized via catechol. Thus, cross-feeding by stenotrophomonads on catechol, or some other naphthalene degradation intermediate, appears to be likely. Although the results of SIP compared with those of cultivation experiments clearly demonstrate that SIP is an efficient method for describing microbial food webs involved in the complete degradation of a substrate, it is difficult to distinguish between primary utilizers of the substrate and cross-feeders. Cultivation could be an efficient supplementary method to SIP which aids in distinguishing between primary substrate utilizers and cross-feeding populations.

## Conclusions

Although pseudomonads have been previously associated with the degradation of PAHs, our study reported an apparent dominance of pseudomonads in the sequestration of carbon from naphthalene. The culture-based component of this study demonstrated the extensive degradation capacities of *P. veronii* and *C. testosteroni*, spanning the seven most abundant PAHs found in the sediment studied. In conclusion, we demonstrated that natural microflora in the sediment had bioremediation potential, and upon delivery of oxygen initially very low abundant indigenous bacteria were capable of metabolizing naphthalene and potentially other PAHs.

## Funding

Financial support was provided by grants awarded to OU from the Czech Science Foundation (no. 13-20414P) and the Czech Ministry of Education, Youth and Sports (no. LH14004). JJ also acknowledges the support provided by J. E. Purkyne's fellowship and the long-term development program RVO 61388971.

### Conflict of interest statement

The authors declare that the research was conducted in the absence of any commercial or financial relationships that could be construed as a potential conflict of interest.
